# Effect of propofol and etomidate on normoxic and chronically hypoxic pulmonary artery

**DOI:** 10.1186/1471-2253-6-2

**Published:** 2006-03-03

**Authors:** Nazinigouba Ouédraogo, Boutchi Mounkaïla, Huguette Crevel, Roger Marthan, Etienne Roux

**Affiliations:** 1 Laboratoire de Physiologie Cellulaire Respiratoire, Université Bordeaux 2, Bordeaux, F-33076 France; Inserm, E356, Bordeaux, F-33076, France; 2 UFR/SDS Université de Ouagadougou, Burkina Faso, France

## Abstract

**Background:**

Chronic alveolar hypoxia results in sustained arterial constriction, and increase in pulmonary vascular resistance leading to pulmonary artery hypertension (PAHT). The aim of this study was to investigate the effect of propofol and etomidate on pulmonary artery (PA) reactivity in chronically hypoxic (CH) rats, a model of pulmonary arterial hypertension (PAHT), in normoxic animals, and human PA.

**Methods:**

CH rats were maintained 14 days at 380 mmHg pressure in a hypobaric chamber. Human tissue was retrieved from histological lung pieces from patients undergoing resection for carcinoma. Cumulative concentrations of anaesthetics were tested on isolated vascular rings precontracted with phenylephrine (PHE) or 100 mM KCl. Statistical comparisons were done by ANOVA, followed, when needed, by Student *t *tests with Bonferroni correction as *post-hoc *tests.

**Results:**

In normoxic rat PA, maximal relaxation (R_max_) induced by etomidate and propofol was 101.3 ± 0.8% and 94.0 ± 2.3%, respectively, in KCl-precontracted rings, and 63.3 ± 9.7% and 46.1 ± 9.1%, respectively, in PHE-precontracted rings (n = 7). In KCl-precontracted human PA, R_max _was 84.7 ± 8.6 % and 66.5 ± 11.8%, for etomidate and propofol, respectively, and 154.2 ± 22.4 % and 51.6 ± 15.1 %, respectively, in PHE-precontracted human PA (n = 7). In CH rat PA, the relaxant effect of both anaesthetics was increased in PHE-precontracted and, for etomidate only, in KCl-precontracted PA. In aorta, CH induced no change in the relaxant effect of anaesthetics.

**Conclusion:**

Propofol and etomidate have relaxant properties in PA from human and normoxic rat. The relaxant effect is specifically accentuated in PA from CH rat, mainly via an effect on the pharmacomechanical coupling. Etomidate appears to be more efficient than propofol at identical concentration, but, taking into account clinical concentrations, etomidate is less potent than propofol, which effect was in the range of clinical doses. Although these findings provide experimental support for the preferential use of etomidate for haemodynamic stability in patients suffering from PAHT, the clinical relevance of the observations requires further investigation.

## Background

Chronic hypoxia (CH) occurs in people living at high altitude and in children who suffer congenital heart disease with left to right shunt; it is also a main characteristic of chronic obstructive pulmonary disease, a major cause of death that affects a significant proportion of adults. Chronic alveolar hypoxia results in sustained arterial constriction, an increase in pulmonary vascular resistance leading to pulmonary artery hypertension (PAHT). At last, PAHT is responsible for right ventricular failure, which may end in death [1,2]. Patients with PAHT may undergo anaesthesia, i. e., for cardiac surgery or heart catheterisation. During general anaesthesia of such patients, variations in cardiac and pulmonary flows may lead to dangerous increase or decrease of systemic or pulmonary pressures. Such variations during catheterism would make the procedure useless. It is then important to consider the effects of the anaesthetics on vascular responsiveness.

The effect of etomidate on vascular reactivity has been poorly investigated. Early clinical studies showed that anaesthesia with etomidate induces little or no change in both pulmonary and systemic arterial pressure [3,4]. Murday *et al*. [5] had observed an increase in pulmonary vascular resistance and a decrease in systemic vascular resistance in patients undergoing cardiac surgery, whereas Shapiro *et al *evidenced a decrease in mean arterial pressure [6]. In experimental studies, etomidate has been shown to inhibit relaxant responses in canine pulmonary arteries [7]. Experiments on isolated rat lung indicated that etomidate is a direct pulmonary vasoconstrictor [8]. The effect of etomidate on systemic and pulmonary vasculature remains hence unclear.

Propofol has become a widely used general anaesthetic during the last decade and was studied in many clinical trials. However, its effect on pulmonary vasculature is unclear. In some clinical trial, propofol altered neither pulmonary mean arterial pressure nor pulmonary vascular resistance, though it decreased systemic vascular resistance [9], whereas in other studies it was shown to decrease both pulmonary arterial pressure and pulmonary arterial resistance [10]. Experimental studies on rats isolated lungs showed a direct vasodilatant effect of propofol [8], and a relaxant effect was also evidenced on rat isolated pulmonary arterial rings [11,12]. By contrast, it has been shown in dogs and canine isolated pulmonary arterial rings that propofol potentiates phenylephrine-induced vasoconstriction [13,14]. As for etomidate, the effect of propofol on pulmonary artery remains hence highly controversial.

Most of the experimental studies have been performed on normal arteries. However, PAHT is associated with morphological and functional changes of pulmonary artery, mainly hypertrophy and hyperplasia of smooth muscle cells, and altered vascular reactivity and Ca^2+ ^homeostasis [15,16]. These functional and morphological alterations have been noted both in small intralobar PA and in main PA, indicating that main PA can be used as a relevant model for the study of pulmonary vasomotricity in CH conditions [17-22]. We have hence assessed the effects of propofol and etomidate on main pulmonary artery reactivity of chronically hypoxic (CH) rats, a model of PAHT, *versus *normoxic rat and human isolated pulmonary arteries. Since chronic hypoxia has differential effects on the systemic *versus *the pulmonary vasculature, we compared the results obtained in rat pulmonary artery with those obtained in systemic thoracic vasculature, i. e., thoracic aorta, in order to determine if the changes in the effect of anaesthetics observed in pulmonary artery were specific.

## Methods

### Chronic hypoxia protocol

The animals used in this study were treated and sacrificed in accordance with national guidelines, and the protocol accepted by local animal experimentation committee. Male Wistar rats, 8–10 weeks old, were exposed to a simulated altitude of 5500 m (barometric pressure 380 mmHg) in a well-ventilated, temperature-controlled hypobaric chamber for 14 days, as previously described [23,24]. Such a protocol is classically used to generate PAHT [18,19,25,26]. In previous studies, our laboratory has characterized this model of hypobaric hypoxia-induced PAHT and shown that this protocol consistently increases mean pulmonary arterial pressure, vessel wall thickness and produces right ventricular hypertrophy evidenced by an increase in the ratio of right ventricle to left ventricle + septum weight (RV/LVS) [15,23,27-30]. Normoxic rats were kept under similar conditions but not in the hypobaric chamber.

### Tissue preparation

Rat pulmonary arteries and aorta from normoxic and hypoxic animals were obtained as follows: for each experiment, a rat was killed by cervical dislocation. The heart and lungs were removed en-bloc, and the extrapulmonary artery and the thoracic part of the aorta were rapidly dissected out. From each specimen, 3 rings 3–4 mm in length were obtained from the main, left and right extrapulmonary artery, and 3 rings of similar length from the thoracic portion of the aorta.

Human bronchial rings were obtained from lung pieces collected for histological examination following resection for carcinoma. Specimens were selected from 7 patients for whom no sign of pulmonary hypertension appeared after pulmonary radiology and clinical examination. Patients were 60.6 ± 4.1 years old. Their lung function was within the normal range: mean forced expiratory volume in 1 second (FEV_1_) was 84.4 ± 6.4% of predicted values and mean partial O_2 _pressure was 83.7 ± 3.3 mm Hg. Quickly after resection, segments of pulmonary arteries (3^rd ^to 5^th ^generation; 3–5 mm in internal diameter) were carefully dissected from a macroscopically tumour-free part of each of the histological pieces and transferred to the laboratory in an ice-cold physiological saline solution. Segments were then cut into rings measuring about 4–5 mm in length for isometric contraction measurements. Use of human tissues was performed according to national guidelines, in compliance with the Helsinki Declaration. Since tissues were obtained incidentally to patient surgery and discarded by the histological pathologist, specific ethical approval of the protocol was not required by French laws.

### Isometric tension measurement

Isometric tension was measured in intact, i. e., with endothelium, vessel rings that were mounted between two stainless steel clips in vertical 20 ml organ baths of a computerized isolated organ bath system (IOX, EMKA Technologies, Paris, France) previously described [15,24]. Baths were filled with Krebs-Henseleit (KH) solution (composition given below) maintained at 37°C and bubbled with a 95% O_2_-5% CO_2 _gas mixture. The upper stainless clip was connected to an isometric force transducer (EMKA Technologies). Tissues were set at optimal length (Lo) by equilibration against a passive load of 1.5 g in rat aorta, normoxic human and rat pulmonary arteries, and 2.5 g in CH rat pulmonary arteries, as determined for these types of preparation in control experiments (data not shown).

The relaxant effect of cumulative concentrations of etomidate and propofol on precontraction to phenylephrine and KCl was assessed as follows. At the beginning of each experiment, prior to exposure to anaesthetics, a contraction was elicited by either a hyperpotassic extracellular solution containing 100 mM KCl or 10^-6 ^M phenylephrine (PHE). According to the Nernst equation, 100 mM KCl depolarises the membrane potential close to -10 mV, which opens the voltage-operated Ca^2+ ^channels and thus activates the electromechanical coupling. PHE is an α_1_-adrenergic agonist that binds to G protein-coupled receptor and acts mainly via InsP_3 _production and Ca^2+ ^release from intracellular stores, the so-called pharmacomechanical coupling.10^-6 ^M PHE induces an inframaximal contractile response, as determined from a cumulative-concentration response curve to PHE in rat aorta and pulmonary artery (n = 4, data not shown). Upon KCl or PHE administration, when the maximal contraction was obtained, propofol or etomidate was added to the vessel rings in cumulative half-log increments from 10^-6 ^to 10^-3 ^M. The ring tension was measured when the response stabilized, i.e., after an equilibration time about 15 min, and expressed as a percentage of the maximal initial contraction of that ring. To avoid any bias due to time-dependent change in tension, the anaesthetic-induced relaxation was normalized to a paired temporal ring, i.e., a ring experimented simultaneously in similar conditions but without exposure to the anaesthetics.

### Chemicals and drugs

PHE were purchased from Sigma (Saint Quentin Fallavier, France). Propofol (Diprivan^®^, Zeneca laboratories, Cergy France) and etomidate (Hypnomidate^®^, Janssen laboratories, Boulogne Billancourt, France) were obtained from their clinically used presentations. We verified that the vehicle of each of the drugs had no effect *per se *on contractile responses up to the maximal concentration used in the present experiments, *i. e*., 1.65%. Normal KH solution contained (in mM): 118.4 NaCl, 4.7 KCl, 2.5 CaCl_2_.2H_2_O, 1.2 MgSO_4_.7H_2_O, 1.2 KH_2_PO_4_, 25.0 NaHCO_3_, 11.1 D-glucose, pH 7.4. For KCl-induced contraction, KCl was substituted to NaCl for the desired concentrations, in order to keep the osmotic pressure constant.

### Analysis of results and statistics

The relaxation induced by each concentration of anaesthetic was expressed as a percentage of the contractile response of the paired temporal control. The concentration-dependent relaxation curves were then fitted by a non-linear Boltzman equation used to determine the concentrations of anaesthetics that reduced the maximal contraction by 50% (IC_50_) and by 30% (IC_30_), reported as negative logarithm (pIC_50 _and pIC_30_, respectively), according to Lovren and Triggle [31], and as we previously used in airways [24]. R_max _refers to the maximal relaxation obtained at the maximal anaesthetic concentration (10^-3 ^M).

Each experimental condition was repeated on 6 to 9 different specimens. Data are given as mean ± SEM. Overall cumulative concentration-response curves in control, etomidate- and propofol-exposed rings were compared using ANOVA for repeated measurements. Comparison of pIC_30_, pIC_50 _and R_max _was done by ANOVA, followed, when needed, by Student *t *tests with Bonferroni correction as *post-hoc *tests. Statistical tests were performed using the SPSS^® ^statistical software. Differences were considered significant when *P *< 0.05.

## Results

### Effect of etomidate and propofol on normoxic rat pulmonary arterial rings

**Figure 1:**
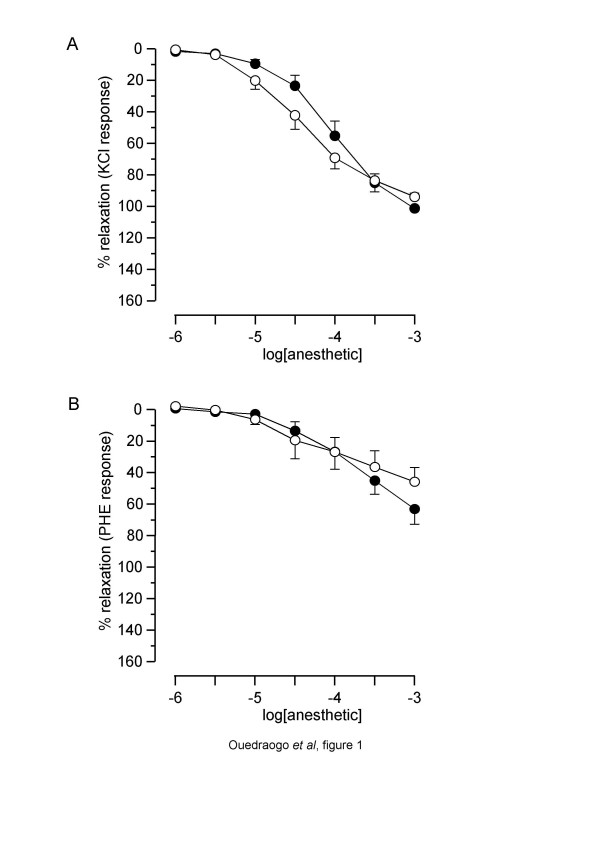
**Relaxant effect of etomidate and propofol on normoxic rat pulmonary arterial rings**. Abscissa: log concentration of anaesthetics (M). Ordinate: isometric contraction (% of the paired temporal control unexposed rings). Full circles: rings exposed to etomidate. Open circles: rings exposed to propofol. A: rings precontracted with 100 mM KCl. B: rings precontracted with 10 ^-6^M PHE. Each symbol is mean value from 5 to 8 rats. Vertical bars are SEM. *P *> 0.05 (overall comparison of etomidate *versus *propofol).

Both etomidate and propofol significantly relaxed precontracted PA rings (figure 1). Overall comparison of the curves showed no difference in the effect of propofol *versus *etomidate in KCl- as well as in PHE-precontracted rings. The relaxant effect of both compounds was greater on KCl-precontracted PA rings, since pIC_30_, pIC_50 _and R_max _were significantly greater in KCl- than PHE-precontracted tissues (tables 1 and 2).

**Table 1:** R_max_, pIC_30 _and pIC_50 _of etomidate on rat pulmonary and aorta and on human pulmonary artery precontracted by KCl and PHE

		R_max _(%control)	pIC_30 _(M)	pIC_50 _(M)	n
*normoxic rat PA*	KCl	101.3 ± 0.8*†	4.32 ± 0.12*‡	4.06 ± 0.13*‡	7
	PHE	63.3 ± 9.7†‡°	3.78 ± 0.19†	3.03 ± 0.44†	7
*human PA*	KCl	84.7 ± 8.6*	3.74 ± 0.12‡	3.46 ± 0.12	7
	PHE	154.2 ± 22.4	3.64 ± 0.16	3.48 ± 0.14	7
*CH rat PA*	KCl	150.0 ± 22.4	4.59 ± 0.11	4.34 ± 0.10	9
	PHE	149.5 ± 19.0	4.55 ± 0.19	4.32 ± 0.19	6
*normoxic rat aorta*	KCl	107.0 ± 3.1	4.16 ± 0.10	3.87 ± 0.09†	9
	PHE	107.0 ± 1.6	4.12 ± 0.14	3.79 ± 0.09†	7
*CH rat aorta*	KCl	112.2 ± 6.6	4.38 ± 0.04	4.11 ± 0.06	8
	PHE	132.7 ± 28.7	4.54 ± 0.16	4.23 ± 0.17	6

Mean maximal relaxation to etomidate (R_max_, % control), and mean 50% and 30% maximal contraction inhibitory concentrations (pIC_50 _and pIC_30_, M) in pulmonary artery (PA) and aorta from normoxic and chronically hypoxic (CH) rat and in human pulmonary artery precontracted with 80 mM KCl (KCl) and 10^-6 ^M phenylephrine (PHE). R_max_, pIC_50 _and pIC_30 _values are mean ± SEM. *P < 0.05 KCl *versus *PHE; †*P *< 0.05 normoxic *versus *HC tissues; ‡*P *< 0.05 rat PA *versus *Human PA; °*P *< 0.05 PA *versus *aorta.

**Table 2:** R_max_, pIC_30 _and pIC_50 _of propofol on rat pulmonary and aorta and on human pulmonary artery precontracted by KCl and PHE

		R_max _(%control)	pIC_30 _(M)	pIC_50 _(M)	n
*normoxic rat PA*	KCl	94.0 ± 2.3*°	4.69 ± 0.14*‡°	4.35 ± 0.14‡°	7
	PHE	46.1 ± 9.1†	3.54 ± 0.26†	1.96 ± 0.76†	7
*human PA*	KCl	66.5 ± 11.8	3.69 ± 0.16‡	3.01 ± 0.30‡	7
	PHE	51.6 ± 15.0	3.33 ± 0.34	3.00 ± 0.49	7
*CH rat PA*	KCl	127.4 ± 15.9	4.83 ± 0.17	4.49 ± 0.17	9
	PHE	90.1 ± 11.7	4.30 ± 0.17	3.86 ± 0.23	6
*normoxic rat aorta*	KCl	79.9 ± 5.3*	4.32 ± 0.10*	3.89 ± 0.14*	9
	PHE	48.1 ± 5.3†	3.53 ± 0.14	2.69 ± 0.38	7
*CH rat aorta*	KCl	101.3 ± 16.7	4.45 ± 0.10	4.15 ± 0.11	8
	PHE	73.1 ± 5.0	4.09 ± 0.24	3.61 ± 0.20	6

Mean maximal relaxation to propofol (R_max_, % control), and mean 50% and 30% maximal contraction inhibitory concentrations (pIC_50 _and pIC_30_, M) in pulmonary artery (PA) and aorta from normoxic and chronically hypoxic (CH) rat and in human pulmonary artery precontracted with 100 mM KCl (KCl) and 10^-6 ^M phenylephrine (PHE). R_max_, pIC_50 _and pIC_30 _values are mean ± SEM. *P < 0.05 KCl *versus *PHE; †*P *< 0.05 normoxic *versus *HC tissues; ‡*P *< 0.05 rat PA *versus *Human PA; °*P *< 0.05 PA *versus *aorta.

### Effect of etomidate and propofol on human pulmonary artery rings

**Figure 2:**
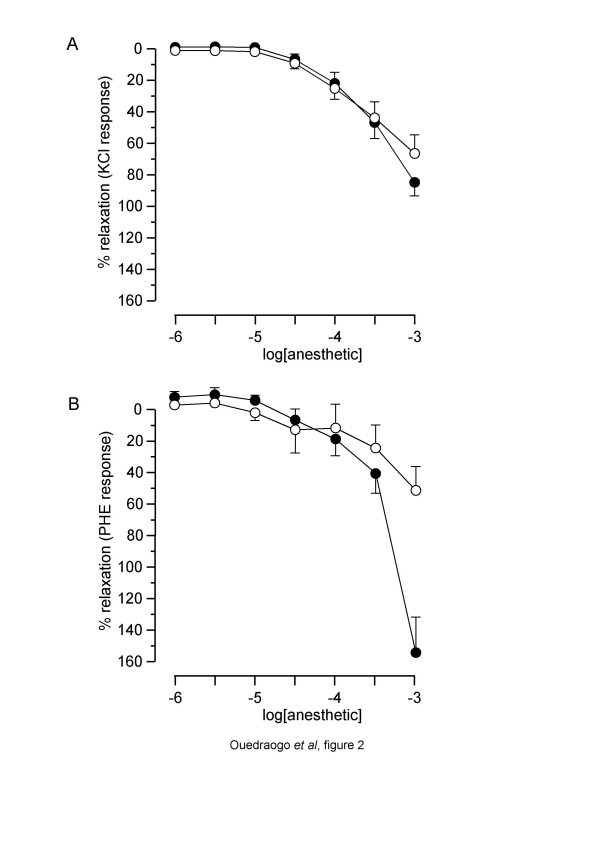
**Relaxant effect of etomidate and propofol on human pulmonary arterial rings**. Abscissa: log concentration of anaesthetics (M). Ordinate: isometric contraction (% of the paired temporal control unexposed rings). Full circles: rings exposed to etomidate. Open circles: rings exposed to propofol. A: rings precontracted with 100 mM KCl. B: rings precontracted with 10 ^-6^M PHE. Each symbol is mean value from 7 specimens. Vertical bars are SEM. *P *> 0.05 (overall comparison of etomidate *versus *propofol).

Overall comparison of the curves showed that propofol and etomidate had a similar concentration-dependent relaxant effect, as shown in fig. 2, despite an apparent greater effect for the highest concentration of etomidate in PHE-precontracted rings. Comparison of human *versus *normoxic rat PA showed that, for both anaesthetics, R_max _was similar or higher in human PA. However, in KCl-precontracted rings, pIC_30 _and pIC_50 _were higher in rat PA (tables 1 and 2).

### Effect of etomidate and propofol on CH rat pulmonary arterial rings

**Figure 3:**
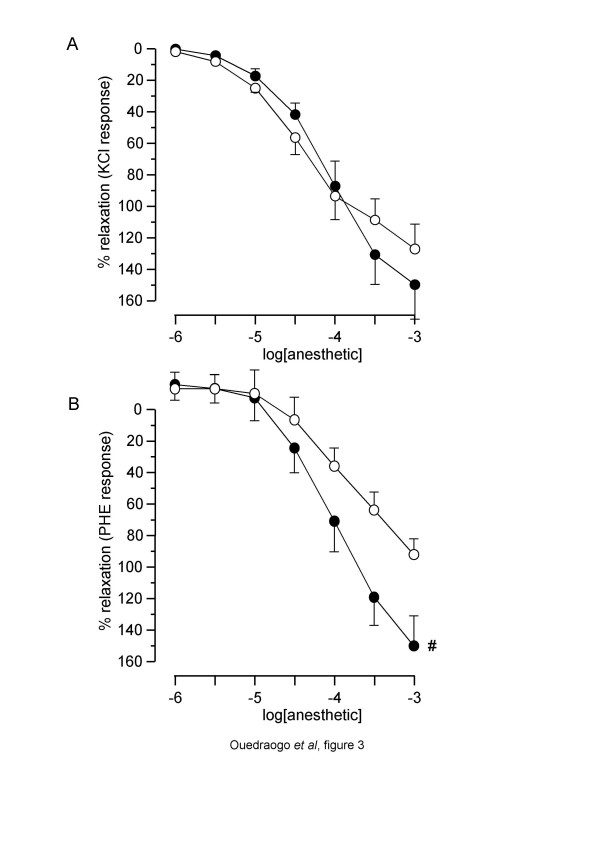
**Relaxant effect of etomidate and propofol on pulmonary arterial rings from chronically hypoxic rats**. Abscissa: log concentration of anaesthetics (M). Ordinate: isometric contraction (% of the paired temporal control unexposed rings). Full circles: rings exposed to etomidate. Open circles: rings exposed to propofol. A: rings precontracted with 100 KCl. B: rings precontracted with 10^-6^M PHE. Each symbol is mean value from 5 to 8 rats. Vertical bars are SEM. **# ***P *< 0.05 (overall comparison of etomidate *versus *propofol).

Both etomidate and propofol significantly relaxed precontracted PA rings from chronically hypoxic rats (figure 3). Overall comparison of the curves showed no difference in the effect of propofol *versus *etomidate in KCl-precontracted rings, whereas etomidate was more potent than propofol in PHE-precontracted PA rings. In contrast with observations on normoxic PA, no significant difference was observed in R_max _and pIC_30 _between KCl- and PHE-precontracted tissues (tables 1 and 2). Comparison between results obtained in PA from normoxic and CH rats showed that in PHE-precontracted rings, R_max_, pIC_30 _and pIC_50 _were higher in CH tissues. In KCl-precontracted rings, though R_max _was greater in CH rings for both anaesthetics, the difference was significant only for etomidate, and no change was observed for pIC_30 _and pIC_50 _(tables 1 and 2).

### Effect of etomidate and propofol on normoxic and CH rat thoracic aorta

**Figure 4:**
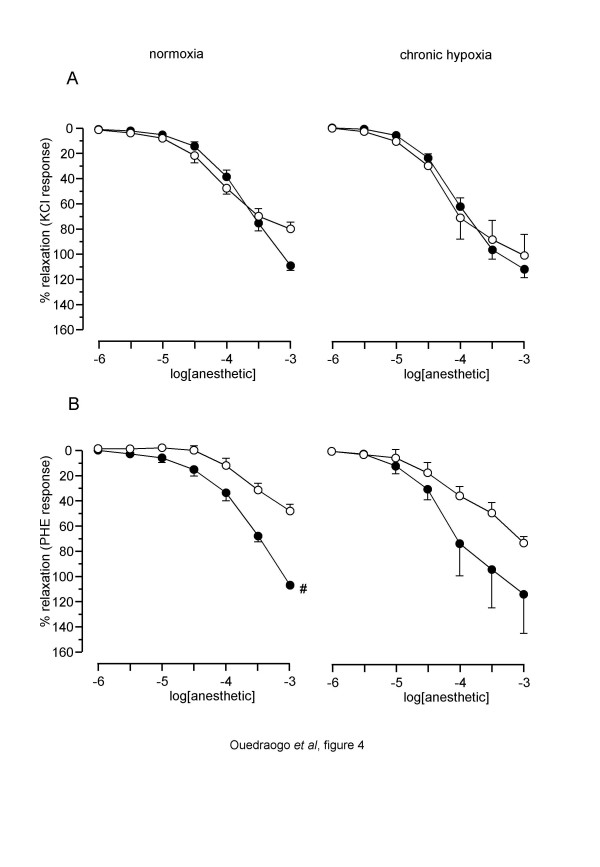
**Relaxant effect of etomidate and propofol on rat aorta rings**. Left panel: aorta rings from normoxic rat. Right panel: aorta rings from CH rat CH Abscissa: log concentration of anaesthetics (M). Ordinate: isometric contraction (% of the paired temporal control unexposed rings). Full circles: rings exposed to etomidate. Open circles: rings exposed to propofol. A: rings precontracted with 100 mM KCl. B: rings precontracted with 10 ^-6^M PHE. Each symbol is mean value from 5 to 8 rats. Vertical bars are SEM. **# ***P *< 0.05 (overall comparison of etomidate *versus *propofol).

In rings from normoxic rats, both propofol and etomidate had a significant relaxant effect on KCl-and PHE-precontracted aortic rings (figure 4). Overall comparison of the curves indicated that etomidate and propofol had a similar effect on KCl-precontracted aorta, but that etomidate was more potent than propofol in PHE-precontracted tissues. Comparisons of R_max _and pIC_30 _and pIC_50 _showed that the relaxant effect of propofol, but not of etomidate, was greater on KCl-precontracted rings (tables 1 and 2). In rings from CH rats, both propofol and etomidate had also a significant relaxant effect on KCl-and PHE-precontracted rings. Overall comparison of CCRC indicated that etomidate and propofol had a similar effect, whatever the contractant agonist. Comparison of R_max_, pIC_30 _and pIC_50 _between normoxic and CH aorta showed that the only difference observed was an increase in the maximal relaxation to propofol in PHE-precontracted CH aorta (tables 1 and 2).

## Discussion

Our results show that propofol and etomidate display a concentration-dependent relaxant effect on pulmonary vasculature. The effect of propofol on PA observed in this study is in accordance with several experimental data that have shown a relaxant effect of propofol on PA [8,11,12] However, some studies have evidenced a contractant effect of propofol on PA [13,14,32,33]. The discrepancy with our study may be due to species differences, since these studies, showing a contractant effect of propofol on PA, were performed in dogs or on canine isolated tissues, whereas we evidenced a relaxant effect of propofol in both rat and human PA. In pulmonary artery, etomidate appeared to have a similar or even greater relaxant effect than propofol. Again, this result is in disagreement with previous studies that have evidenced an antirelaxant effect of etomidate in canine pulmonary artery [34]. In addition to species or tissues differences, it should also be noticed that in these studies the experimental conditions differed from ours and focused on the possible effect of etomidate on relaxant agents, not contractant ones.

Comparison of the R_max _obtained in rat *versus *human pulmonary arteries indicates that both anaesthetics have similar or even greater maximal relaxant effect in human compared to rat tissues. However, it should be noticed that for etomidate as well as propofol, pIC_30 _and pIC_50 _were higher in rat than in human PA precontracted with KCl. This indicates that, though both anaesthetics have a relaxant effect on PA from both species, human PA is less sensitive than rat one to the effect of propofol and etomidate, suggesting small, if any, relaxant effect of etomidate and propofol on normoxic human pulmonary artery at clinical concentrations.

For the two anaesthetics tested, the relaxant effect was greater in CH versus normoxic tissues. Though experiments, for obvious ethical considerations, have not been performed in human tissues from CH hypoxic patients (CH been a usual counterindication for lung surgery), our results suggest that the effect of these anaesthetics on haemodynamics may be greater in patients suffering chronic hypoxia, especially on pulmonary haemodynamics. CH has different effects on systemic and pulmonary vasculature. It has been shown that hypoxia induces systemic vasorelaxation and CH does not induce morphological changes in systemic vasculature [35]. By contrast, hypoxia induces a specific vasoconstriction in pulmonary artery, and CH induces pulmonary hypertension and remodelling [15,16,36]. Our study showed that the enhanced relaxant effect of etomidate and propofol in tissues from CH animals is principally observed in pulmonary artery. This indicates that the changes in anaesthetic sensitivity are not due to chronic hypoxia *per se*, but specifically associated with the CH-induced pulmonary hypertension.

The enhanced effect of the anaesthetics was mainly observed on PHE-precontracted PA. Hence, the increased sensitivity of CH PA is mainly due to an enhanced effect of the anaesthetics on the pharmacomechanical coupling. This suggests that chronic hypoxia modifies the pharmacomechanical coupling of pulmonary artery, in agreement with previous findings on CH-induced calcium signalling in pulmonary arterial smooth muscle cells [15]. Since the pharmacomechanical coupling is activated not only by α-adrenergic stimulation but also by several major physiological vasoactive agonists such as endothelin 1 and angiotensin 2, it is likely that propofol and etomidate may alter the physiological regulation of the pulmonary vasomotricity. Several authors have described an endothelium-dependent effect of propofol and etomidate on pulmonary vascular resistance [32,34]. Since our experiments were performed in rings with intact endothelium, we cannot exclude that the enhanced relaxant effect of these anaesthetics on pulmonary arteries from CH rats may be endothelium-mediated. However, the endothelium-dependent effect of these anaesthetics have been evidenced on ACh-induced relaxation, whereas the effect of propofol and etomidate on pulmonary arteries stimulated by contractile agonists has been shown to be endothelium-independent [8,11,13,14,32,34]. In particular, the effect of propofol, which was shown to be epithelium-dependent when assessed on ACh-induced relaxation [32], appeared to be epithelium-independent when tested on α-adrenergic contraction [11,14], though with opposite consequence in dogs [14] and rats [11], as mentioned above. Since the enhanced effect of both anaesthetics were mainly observed on phenylephrine-precontracted rings, it is therefore likely that this effect may be endothelium-independent.

Though pulmonary artery and aorta rings used in this study were from chronically hypoxic animals, the experimental conditions for isometric measurements were not hypoxic, rather hyperoxic, since tissues were bubbled with 95% O_2 _and 5% CO_2_. Since anesthetized patients are usually ventilated with hyperoxic gas mixture, our experimental conditions remain, nevertheless, clinically relevant.

Blood concentrations of propofol and etomidate, following clinical injection, are about 3–15 μg.mL^-1 ^and 0.5–1,6 μg.mL^-1^, respectively [37-41]. This corresponds to 10^-5^-10^-4 ^M for propofol concentration, and about 2–6.10^-6 ^M for etomidate. At these concentrations, below IC_30 _values, etomidate has no significant relaxant effect in normoxic tissues and, though enhanced, small effect on CH ones. This may explain why clinical studies have generally not concluded to a relaxant effect of etomidate on PA [5,8], and suggests a small, if any, effect of etomidate on pulmonary haemodynamics in CH patients. By contrast, clinical concentrations of propofol may be in the same range of IC_30 _values. This may explain why some authors have concluded that propofol infusion at clinical doses decreases pulmonary arterial pressure and pulmonary arterial resistances [10]. Moreover, since the relaxant effect was increased in CH rat pulmonary arteries, the effect of propofol on pulmonary haemodynamics may be higher in CH subjects. Hence, our study provides experimental support for the preferential use of etomidate for the maintenance of haemodynamic stability in patients suffering from PAHT. However, one should be cautious in extrapolating *ex vivo *data to *in vivo *conditions, in particular because of the high protein binding of these compounds which decreases their free concentrations and hence their biological effect [42].

## Conclusion

In conclusion, our study demonstrates that in normoxic rats etomidate and propofol have a relaxant effect on pulmonary artery, acting mainly on the electromechanical coupling and, to a lesser degree, on the pharmacological coupling. A relaxant effect was also observed in human pulmonary artery, though human PA appears to be less sensitive to the anaesthetics than rat one. The effects of both anaesthetics were greater on PA from an animal model of hypoxia-induced pulmonary hypertension. This enhanced relaxant effect was specific to PA and was mainly seen on the pharmacomechanical coupling. Etomidate appears to be more efficient than propofol at identical concentration. However, comparison that take into account difference between etomidate and propofol concentrations used at clinical doses indicates that etomidate is less potent than propofol, which may have an effect on pulmonary haemodynamics, especially in subjects suffering CH and PAHT. Although these findings provide experimental support for the preferential use of etomidate in patients suffering from PAHT, the clinical relevance of the observations requires further investigation.

## List of abbreviations

CCRC: cumulative-concentration response curve

HC: chronic hypoxia

IC_50_: concentration that reduces the maximal contraction by 50%

IC_30_: concentration that reduces the maximal contraction by 30%

KH: Krebs-Henseleit

PA: pulmonary artery

PHE: phenylephrine

pIC_50_: negative logarithm of IC_50_

pIC_30_: negative logarithm of IC_30_

R_max_: maximal apparent relaxation

## Competing interests

The author(s) declare that they have no competing interests.

## Authors' contributions

NO participated in the conception of the study and its design, the experiments in rat and human tissues, participated in the analysis of the data, carried out the statistical analysis, and helped the draft of the manuscript. BM participated in the experiments in rat tissues and the analysis of data. HC participated in the contractile experiments in rat and human tissues. RM participated in the design of the study and helped the draft of the manuscript. ER participated in the conception of the study and its design, helped in statistical analysis and drafted the manuscript.

## Pre-publication history

The pre-publication history for this paper can be accessed here:


